# Protocol for assembling micro- and nanoparticles in a viscous liquid above a vibrating plate

**DOI:** 10.1016/j.mex.2018.09.008

**Published:** 2018-09-28

**Authors:** Soheila Shabaniverki, Sarah Thorud, Jaime J. Juárez

**Affiliations:** Iowa State University, Department of Mechanical Engineering, 2529 Union Drive, Ames, IA, United States

**Keywords:** Protocol for assembling micro- and nanoparticles in a viscous liquid above a vibrating plate, Directed assembly, Acoustophoresis, Colloids

## Abstract

In this protocol, we demonstrate the use of a vibrating plate to drive the assembly of micro- and nanoparticles as an approach to high-throughput, large-scale directed assembly in a viscous liquid. Vibration drives the assembly of glass bead microparticles and iron oxide nanoparticles in contact with water over an area of 6400 mm^2^. We use a scaling analysis to show that there is a competition between acoustic radiation force and vibration-generated fluid flow in a viscous medium, which determines particle transport characteristics. For assembly in a viscous liquid, we find close agreement between the observed experimental results when compared to a numerical solution of the 2D wave equation that describes plate displacement. This model indicates that microparticles migrate along displacement gradients towards displacement anti-nodes where the magnitude of displacement is maximum. We also observe that nanoparticles migrate toward displacement nodes where the magnitude of displacement is zero.

• Cost-effective directed assembly technique without the need for microfabrication facilities
• Large-scale assembly produces heterogeneously ordered structures on a vibrating substrate

## Graphical abstract

**Figure:**
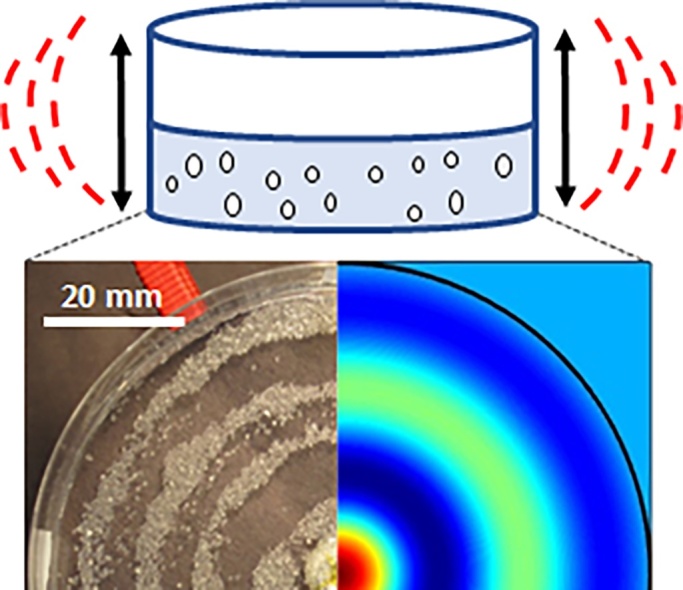

**Specifications Table**

**Table:** 

Subject area	• *Engineering*
More specific subject area	*Directed self-assembly*
Method name	*Protocol for assembling micro- and nanoparticles in a viscous liquid above a vibrating plate*
Name and reference of original method	S. Shabaniverki, S. Thorud and J. J. Juárez, Vibrationally directed assembly of micro- and nanoparticle-polymer composites, Chemical Engineering Science, DOI: https://doi.org/10.1016/j.ces.2018.06.068. E. Chladni, Entdeckungen über die Theorie des Klanges, Nabu Press, 1787.

## Introduction

The directed assembly of micro- and nanoparticles provide a pathway for the fabrication of materials that support a variety of emerging applications for functional surfaces [1], smart material actuator elements [2], electrically conductive networks [3], and tunable biomechanical constructs [4]. Tuning the alignment of these particles during assembly can influence the underlying material properties [5]. The capacity for particle arrangement to influence bulk properties is characteristic of bottom-up material design, where the desired material property is obtained by directly manipulating the assembly process.

In order to meet the increased demand for directly assembled materials for emerging applications, it is critical to have a variety of assembly techniques available to handle a wide variety of materials. The use of transport mechanisms generated by non-uniform electric fields [6], such as dielectrophoresis or electroosmotic flow, can be used to directly assemble particles into ordered structures. Similar to the electric fields, non-uniform magnetic fields can also drive the transport of particles into ordered structures [7]. While these fields are capable of forming a wide array of microstructures, these approaches are limited to particles with specific properties or by the size scale of the device used for assembly (∼25 mm^2^ or less for electric fields [8]).

The protocol presented here forms the basis for our published work on the assembly polymer-particle based composite materials [9]. This protocol was based on the traditional physics experiment first described by Ernst Chladni, where small particles on a vibrating substrate assemble into different patterns depending on the input frequency of an acoustic wave [10]. While our published work focused on assembly in a polymer solution, this protocol describes assembly of particles in water.

The protocol described here offers several advantages from a technical and pedagogical standpoint. This platform is capable of assembling particles on a large scale (∼6400 mm^2^), which represents a significant scale up (∼256 times) in comparison to microfabricated devices utilizing external fields (e.g., electric fields). The reliance on portable off-the-shelf supplies and equipment represent a significant cost-savings as this system does not rely on facilities for microfabrication and that assembly can be done almost anywhere. The protocol we describe here could be used as a pedagogical tool to incorporate directed assembly as part of the curriculum for a multidisciplinary course in nanotechnology [11,12].

## Method details

An alternative to electric or magnetic fields is the use of physical substrate vibration to drive particle assembly. Chladni was the first to describe the assembly of small particles on a vibrating substrate in 1787 [10]. The mechanical strain placed on the substrate by localized flexing can lead to pressure waves that propagate through a medium as a surface standing acoustic wave (SSAW) [13]. The acoustic radiation produced by these pressure waves interact with particles in a medium to transport them to acoustic nodes or antinodes depending on particle-medium properties [14].

The effect of transporting particles in external acoustic fields, acoustophoresis, has been used to assemble a variety of microparticles [15,16], nanoparticles [17,18] and biological samples [19,20]. The acoustic field can act on a variety of material combinations so long as a density and compressibility difference between the assembling particles and the medium exists. Acoustic fields can be generated using off-the-shelf components [21] with minimal use of microfabrication and can act on areas (∼1000 mm^2^) that are larger than other directed assembly approaches [22].

In the protocol presented in this article, glass bead microparticles and iron oxide nanoparticles were assembled on a large scale (∼6400 mm^2^) vibrating substrate in water. The input vibration generates two dimensional standing waves which cause particle migration in the solution. The frequency and amplitude were varied to test the self-assembly of the particles. The amplitude of the vibrational waves was varied to test if it affected the particle movement speed or the number of particles moving toward patterns (as opposed to remaining stationary). The frequency was varied to observe what patterns or microstructures could assemble given the initial parameters of the experimental set-up.

Materials

• Glass microparticles with size distribution of 425–600 μm were sourced from Sigma-Aldrich (product number G8772).
• Iron (II, III) oxide nanoparticle with size distribution between 50–100 nm was sourced from Alfa Aesar (product number 47,141).
• The micro- and nanoparticles are dispersed in deionized water, which was sourced from an ARIES High Purity Water System with a 0.2 μm filter.

Equipment

• A function generator (Vizatek, model number 01VZMFG2120) is connected to a mechanical wave drive (Pasco, part number SF-9324) to introduce an oscillatory vibration to the substrate.
• A 95-mm diameter polystyrene petri dish is used as a substrate to assemble glass beads (Fisher Scientific, catalog number FB0875714G).
• Images of microparticles pattern were captured using a Canon PowerShot SX530 HS at 16-megapixel resolution.

## Experimental procedures

**Fig. 1:**
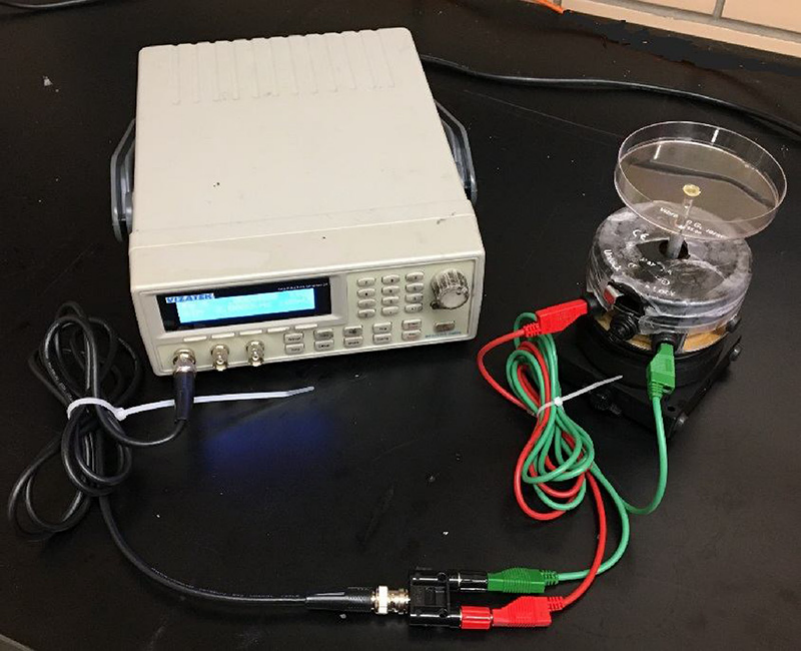
The experimental setup used to assemble particles with vibration. A function generator (left) is connected to the mechanical wave driver (right) with a banana connector. A petri dish with diameter of 95 mm is attached to the mechanical wave driver and used as the substrate for assembly.

A polystyrene petri dish served as a substrate for holding the dispersions during vibrational assembly. Epoxy was used to attach a screw centered on the bottom of the petri dish. After giving the epoxy 24 h to cure, the petri dish was attached to the moving arm of the wave generator which produces sinusoidal waveform. The prepared dispersion was added to the petri dish. Fig. 1 shows the experimental setup and components used to vibrate substrate and assemble particles.

After the substrate is attached to the moving arm of the mechanical wave generator, we add 20 mL of water to the petri dish. For the microparticle experiments, one and a half grams of glass beads were added to 20 mL of water in the petri dish without modification to prepare a microparticle dispersion in a viscous fluid. Nanoparticle dispersions were prepared by adding one gram of iron oxide to 20 mL of water.

For these experiments, we varied input voltage amplitude (2 V–6 V) and frequency (100 Hz – 2000 Hz). When the petri dish is mechanically driven by the wave generator by these inputs, we observe that the particles migrate in solution. The mechanical input produces a two-dimensional standing wave within the substrate as a result of the vibration. The glass microparticles migrate to displacement anti-nodes, where they assemble into well-defined clusters within 40–50 s after the start of the experiment. In order to document our results, we positioned a tripod with a camera directly above the petri dish to image the patterns that formed at different frequencies.

## Scaling analysis

The vibration of our petri dish substrate generate patterns within the substrate caused by dish motion, which arise because of stresses caused by substrate flexing [23]. The patterns formed by substrate flexing are referred to as vibrational modes. When the input causing substrate vibration is steady, the vibrational modes generate patterns that are associated with specific patterns that are referred to as natural frequencies [24]. Our experiments are conducted in DI water and, as a result of the viscous nature of our medium, we expect that the natural frequencies will occur at lower values when compared to similar systems where air is the medium [25]. We compare our experimental results to a numerical solution of the two-dimensional wave equation in MATLAB to identify the vibrational modes in our sample. The solution of the two-dimensional wave equation is expressed as [26],

(1) $$A\left(r,\theta \right)/A_{o}=J_{m}\left(\alpha_{mn}\frac{r}{R}\right)cos(m\theta )$$

Where *A_o_* is the peak amplitude of the plate vibration, *J_m_* is a Bessel function of the first kind with order m, *α* is the *n*th zero of a Bessel function of order *m*, and *R* is the plate radius. The position on the plate is expressed in terms of polar coordinates, *r* and *θ*, with the origin of our coordinate system located at the center of the center of the petri dish. The parameters m and n represent the different modes of vibration in our system. By adjusting *m* and *n*, we can identify the dominant modes at different frequencies. The vibration of a substrate with a fluid on top generates a pressure wave with a distribution at the solid-fluid interface, *p_i_*, given by the Helmholtz wave equation [27],

(2) $$\nabla^{2}p_{i}=-\frac{\omega^{2}}{c^{2}}p_{i}$$

where *c* is the speed of sound in the medium above the vibrating plate. We can relate the fluid velocity at the substrate boundary to the pressure distribution as [28],

(3) $$\frac{\partial v}{\partial t}=-\frac{1}{\rho_{f}}\nabla p_{i}$$

(4) $$v=\frac{\partial w}{\partial t}$$

where *ρ_f_* is the density of the fluid above the plate. Eqs. (3) and (4) are first-order hydrodynamic equations that assume density and pressure are independent of time [29]. This approximation is reasonable in that our protocol is unlikely to generate a pressure field of sufficient magnitude that compressibility is a factor.

When we combine Eqs. (3) and (4) with the following identity for time-dependent plate displacement, *w* = *W(r,θ)·*cos*(ωt)*, we find that the pressure gradient at the interface is,

(5) $$\nabla p_{i}=\rho_{f}\omega^{2}w$$

Substituting the pressure gradient term in Eq. (5) into the Laplacian term from Eq. (2) allows us to relate the pressure at the interface with the gradient of displacement,

(6) $$p_{i}=-\rho_{f}c^{2}\nabla w$$

Near the solid-fluid interface, the pressure distribution produces a primary acoustic force that acts on particles in the dispersion and scales with the gradient of pressure [30,31],

(7) $$F_{ac}=\frac{1}{2}\pi V_{p}\beta_{m\;}\Phi \nabla p_{i}^{2}$$

where β_m_ = 1/ρ_f_c^2^ is the medium compressibility factor and Φ, known as the acoustic contrast factor, is expressed as,

(8) $$\Phi \;=\;\frac{{5\rho }_{s}-2\rho_{f}}{{2\rho }_{s}+\rho_{f}}-\frac{\beta_{s}}{\beta_{f}}$$

Based on this analysis, we expect that the acoustic radiation force and vibration-induced fluid flow to each play a role in determining the transport characteristics of assembling particles. In order to understand the competition between these two mechanisms on our results, we introduce a scaling analysis. The physical parameters that play a role in determining which mechanism is significant can be written in matrix form as,

(9) $$\Pi =\left[\begin{array}{ccc} & \rho_{f} & \begin{array}{ccc} v & a & \begin{array}{cc} \mu & F_{ac} \end{array} \end{array}\\ M & \;1 & \begin{array}{ccc} 0 & 0 & \begin{array}{cc} \;1 & 1 \end{array} \end{array}\\ \begin{array}{c} L\\ T \end{array} & \begin{array}{c} -3\\ \;0 \end{array} & \begin{array}{c} \begin{array}{ccc} 1 & 1 & \begin{array}{cc} -1 & 1 \end{array} \end{array}\\ \begin{array}{ccc} 1 & 0 & \begin{array}{cc} -1 & 1 \end{array} \end{array} \end{array} \end{array}\right]$$

where a is particle radius, μ is the fluid viscosity and the letters *M*, *L* and *T* represent the dimensions of mass, length and time, respectively. Selecting fluid density, fluid velocity and particle radius as repeating variables, the first dimensionless group that we identify is,

(10) $$\Pi_{1}=\frac{\mu }{\rho_{f}av}$$

which compares the viscous forces to the inertial forces experienced by the particles in a viscous medium above a vibrating substrate (i.e., inverse Reynolds number of the particle). In order to approximate the fluid velocity for this scaling model, we examine the time-dependent displacement of the plate, *w* = *A(r,θ)·*cos*(ωt)*. The rate at which this displacement changes with time is given by, *dw/dt = v* = −*A(r,θ)·ω ·*sin*(ωt)*. By the no-slip condition, the velocity of the fluid should be equal to the velocity of the plate at the fluid-solid plate interface. Based on this model, the magnitude of this velocity is *v_max_* = *A_o_ω*, which we use as a scaling approximation for fluid velocity. This approximation is consistent with a scaling approximation for vibrationally excited particles in granular systems [32]. When we introduce this scaling definition for fluid velocity (*v* ∼ *ωA_o_*) to the definition of an acoustically generated boundary layer thickness [33], $\delta =\sqrt{2\mu /\rho_{f}\omega }$, the dimensionless group is re-written as,

(11) $$\Pi_{1}=\frac{\delta^{2}}{2aA_{o}}$$

By re-writing the definition of the first dimensionless group in this form, we are able to compare the characteristic length scale of the acoustic boundary layer that forms in the presence of a vibrationally-induced fluid flow to particle size. This indicates that when a fluid flow is generated by the vibrating substrate, particles that are smaller than the boundary layer thickness are more likely to become entrained in the fluid and move away from regions where the flow is strongest (displacement anti-nodes) and towards regions where the flow is less significant (displacement nodes).

The second dimensionless group compares the acoustic radiation force to the inertial force experienced by particles in solution,

(12) $$\Pi_{2}=\frac{F_{ac}}{\rho_{f}a^{2}v^{2}}$$

Assuming that the gradient term scales as *R*^−1^ ($\nabla ∼R^{-1}$), displacement scales with amplitude (*w* ∼ *A_o_*), and an acoustic contrast factor of order 1, the acoustic radiation force acting on a particle is approximately,

(13) $$F_{ac}\approx 6.58\frac{a^{3}\rho_{f\;}c^{2}A_{o}^{2}}{R^{3}}$$

Substituting Eq. (13) into Eq. (12) and combining a scaled value for fluid velocity (*v* ∼ *ωA_o_*), we find that the second dimensionless group is,

(14) $$\Pi_{2}=\frac{6.58ac^{2}}{\omega^{2}R^{3}}$$

At sub-micron size scales, particles dispersed in a viscous medium above a vibrating plate are dominated by flow generated by the vibration (Eq. (11)). Particles on these scales become entrained in the fluid flow and are transported toward acoustic displacement nodes. Larger particles are more sensitive to the acoustic radiation force, leading their transport to favor motion toward acoustic displacement anti-nodes (Eq. (14)). The scaling rules presented here are consistent with previously reported transport of microparticles and nanoparticles above a vibrating plate [11].

## Results

**Fig. 2:**
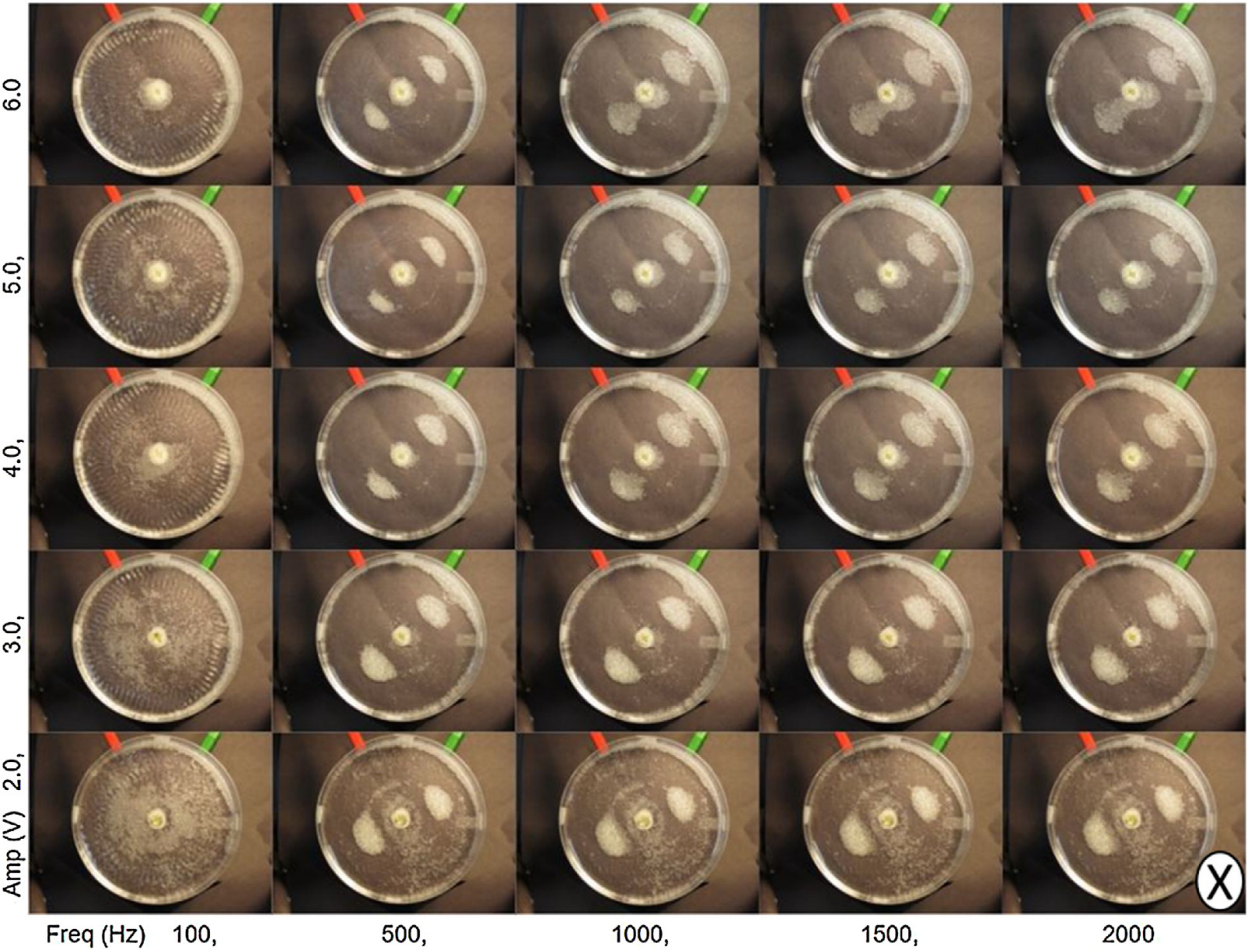
An experimental phase diagram showing how the assembly of glass beads, with a size distribution of 425–600 μm, varies with frequency and amplitude. The circle with X is used to indicate out of place motion.

Voltage amplitude was varied (2 V–6 V) along with frequency (100 Hz – 2000 Hz) in the experimental phase diagram shown in Fig. 2. The diagram demonstrates that amplitude does not affect resonant frequencies. Patterns formed at to within 40 Hz of the frequency, regardless of input voltage. The voltage influences plate amplitude, which affects the rate of particle assembly. Particles moved slowly or not at all at voltages less than 4 V, while the rate increased with voltages higher than 4 V. Plate amplitude affects the number of particles moving toward patterns, as opposed to particles remaining stationary in the dish. When the input voltage is low (2 V), few particles move toward patterns and when the input voltage is high (6 V), most particles in the dish move toward resonant mode patterns.

**Fig. 3:**
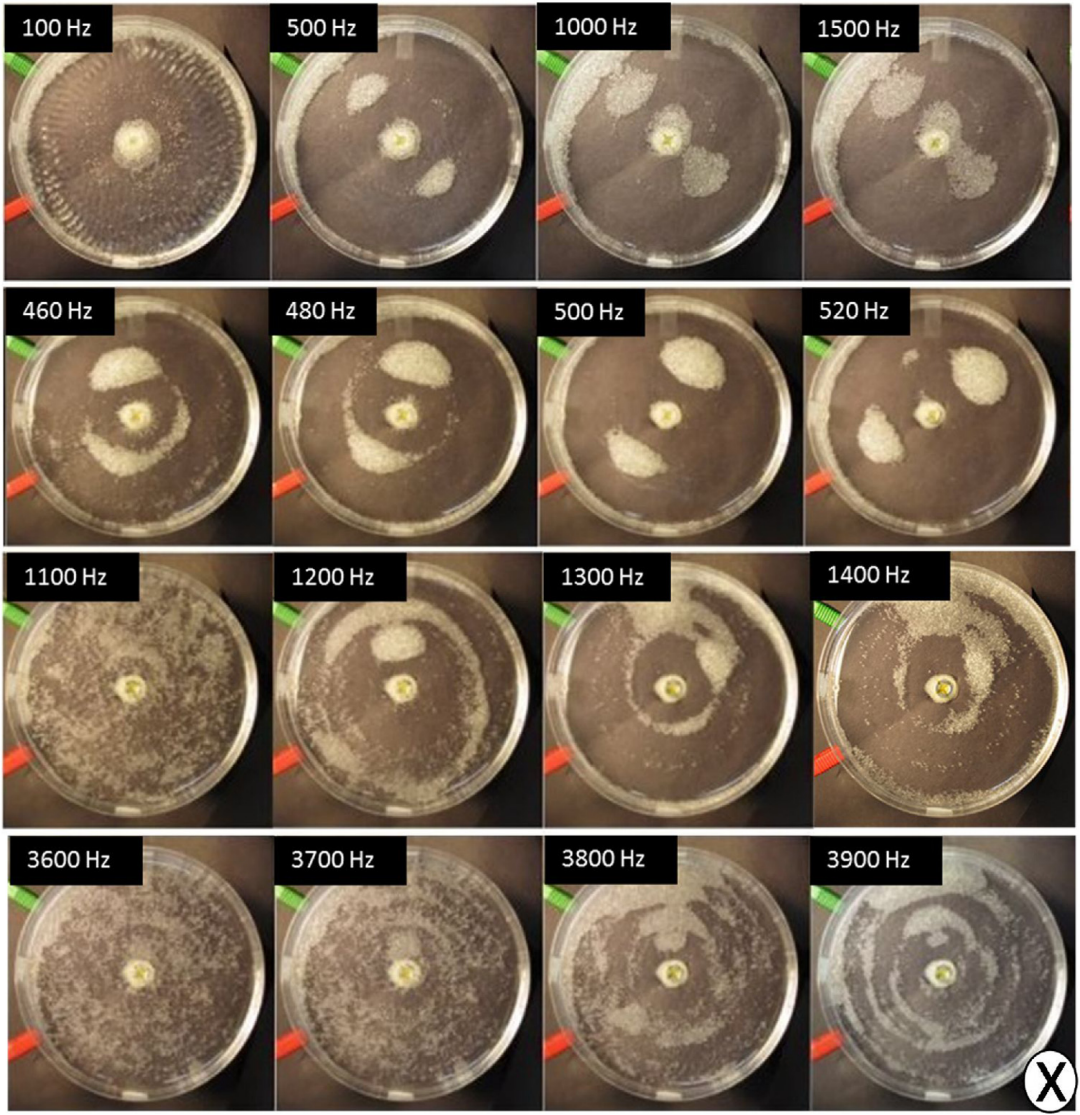
The assembly of glass bead microparticles, with size distribution of 425–600 μm, in contact with DI water into a polystyrene petri-dish, which vibrates at different frequencies. At each frequency, the particles either transition between different modes (1100 Hz, 3600–3700 Hz) or form into assembled structures with a combination of clusters and rings.

Fig. 3 shows the assembly of glass beads in contact with DI water at a voltage of 6 V peak-to-peak. The glass beads are larger (425–600 μm) than the characteristic boundary layer, δ, which ranges between 8 μm (3900 Hz) and 53 μm (100 Hz) for the frequency range examined in Fig. 3. This suggests that the boundary layer has very little effect on assembly. Based on our results in Fig. 2, it is apparent that voltage does not influence the type of assembled pattern, with the same patterns forming at their corresponding frequencies regardless of voltage input. Since voltage and plate amplitude are related parameters, these results are consistent with the scaling parameter from Eq. (14), where frequency is a more significant driver of assembly. The amplitude does appear to affect the rate at which particles assemble, with particles assembling into the patterns at displacement antinodes shown in Fig. 3 at a faster rate at 6 V as compared to the lower voltage cases.

**Fig. 4:**
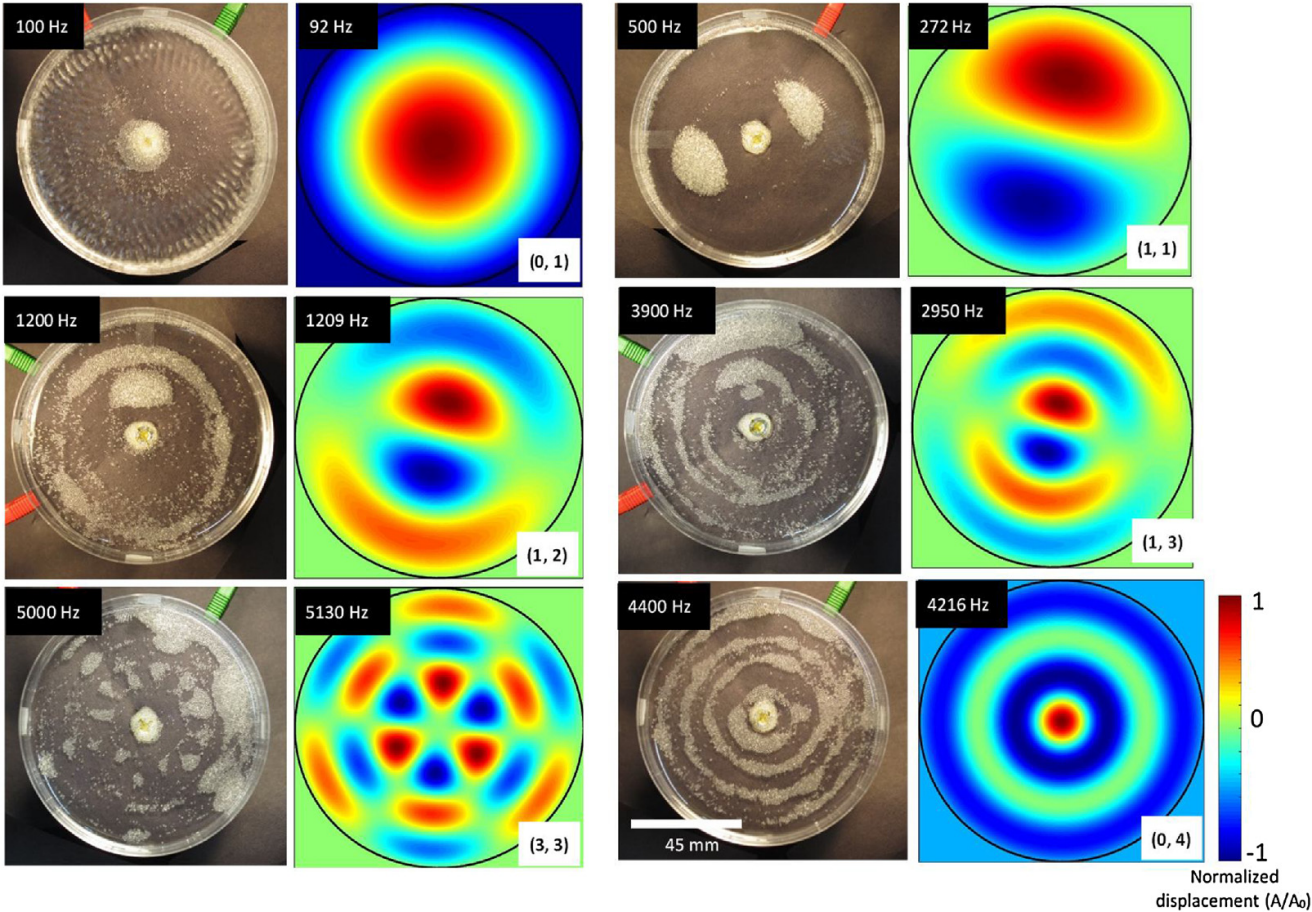
Experimental results for glass bead vibrational assembly at different frequencies and modes of vibration. The expected normalized displacement pattern (A/A_o_) and fundamental frequencies (upper left, contour plots) closely match the observed frequencies (upper left, experimental images) and patterns. These results show that micron size glass beads collect at displacement anti-nodes.

Fig. 4 compares the experimental results for glass bead assembly to a numerical solution of plate displacement based on the plate equation of motion. We arrived at these solutions by varying the vibration modes, m and n, in the simulation. At the lowest frequency we observed for glass beads (100 Hz), the particles migrate to the substrate edge or center as these regions experience the largest degree of displacement. Ripples visible in the fluid surface are indicative of fluid motion during vibration. By comparing the observed pattern to the amplitude distribution, we identified this assembly behavior as being consistent with the lowest observable vibration mode of m = 0 and n = 1 or (0,1).

The next fundamental frequency with a mode of (1,1) is encountered at 500 Hz with the formation of two off-center clusters. The first partial ring appears at 1200 Hz with a vibrational mode of (1,2), where we also expect two off-center clusters. The second cluster is likely absent as a result of an unbalanced plate, leading to preferential migration induced by gravity towards one side of the plate. The vibrational mode (1,3) appears at 3900 Hz with the formation of partial rings and clusters. Our results also indicate plate imbalance may be a more significant factor at higher frequencies with bead migration towards one side of the plate. Fully formed rings appear for the first time in our experiments at 4400 Hz, corresponding to the vibrational mode (0,4).

The next vibrational mode appears at 5000 Hz. This frequency value closely correlates with the vibrational mode of (3,3). The experimental results appear to have twice the number displacement nodes clustered around the center (∼11) of the plate than what we expected based on our calculations (6 displacement nodes). The placement of the pinning point in the middle of the substrate, could be slightly off-center. This leads to an unbalanced plate and could also be responsible for slightly different vibrational response than what we expect based on the driving frequency.

**Fig. 5:**
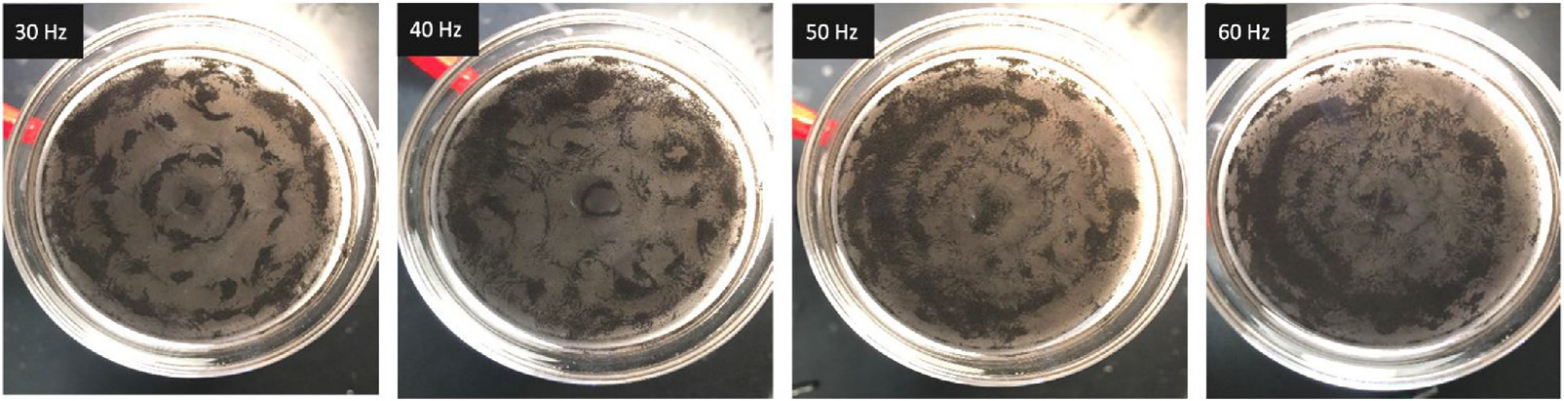
The assembly of iron oxide nanoparticles in contact with DI water into a glass petri-dish which is resonating at four different frequencies, ranging from 30 Hz to 60 Hz.

Fig. 5 shows the assembly of iron oxide nanoparticles in DI water above a vibrating plate. The nanoparticles, with a nominal diameter of 50–100 nm, were found to not assemble into definite patterns. The size scale of these particles is below the expected scale of the boundary layer, which suggests that these particles become entrained in fluid flow. The patterns that do emerge during assembly appear to be broken up by regions without particles. Based an analysis of nanoparticle assembly by acoustophoretic focusing, nanoparticle assembly is highly sensitive to the acoustic response of the medium through the acoustic contrast factor [18]. In our published results [9], the addition of a polymer to the fluid modified the properties of the medium to allow for the assembly of nanoparticles to assemble into microstructures.
